# Skin Diseases and Syphilis

**Published:** 1894-07

**Authors:** 


### SKIN DISEASES AND SYPHILIS.

EDITED BY DR. M. B. HUTCHINS.

### SOME COMMON MISTAKES IN THE TREATMENT OF SYPHILIS.

Dr. George H. Fox read a paper on this subject.* Many physicians, Dr. Fox said, hold to the belief that syphilis is an incurable disease. On the contrary, the disease in every case tends to run a natural course and get well of itself. If a patient suffering from syphilis inherits a sound constitution and takes care of himself, the prognosis is extremely favorable even though no treatment whatever is adopted. With the methods of treatment at our command, no disease furnishes such good results.

Another common mistake arises from the belief that mercury and potassium iodide are practically the only remedies we have at our command in the treatment of syphilis. While they are both very potent remedies, yet complete reliance on them often causes serious injury to the patient. In anemic patients iron should be regarded as an anti-syphilitic remedy. In strumous individuals, cod liver oil is very serviceable. The alleviation of mental anxiety and the adoption of hygienic rules are of the utmost importance in certain cases. The mistake is too frequently made that we treat the disease instead of the patient.

Another fallacy is the belief that a certain definite period of time is required to effect a cure. Some say two years, others three, etc. The course of syphilis varies in different individuals, and the period of treatment must likewise vary, according to the severity of the case. One case of syphilis may require twice as much medicine as another, and the period over which treatment should be extended may be twice as long.

Another common error is that many ills occurring in a syphilitic subject are treated as though they were of syphilitic origin. The fact that a patient has syphilis does not exempt him from non-specific disorders, yet the physician is very apt to jump to the conclusion that such disorders are the result of the syphilis, and to

*Meeting of New York Academy of Medicine April 10, 1894

treat them accordingly. In many cases lesions on the tongue and oral mucous membrane in syphilitics remain unaffected by specific treatment, and the fact should be borne in mind that similar lesions may occur in persons who have not had syphilis, as the result of digestive disturbance. Even if they are syphilitic, such lesions may persist in spite of specific remedies unless the digestive errors are corrected.—*Journal of Cutaneous and Genito-Urinary Diseases*, June, 1894.

---

### MASSAGE IN THE TREATMENT OF PRURIGO.

Eleven cases, seven of a severe form, four milder, have been benefited by this procedure in the hands of R. Hatschek, of Vienna. His method of application is a simple rubbing of the diseased surface, beginning at the distal end, if it is an extremity, and working toward the center. The séances at first last from ten to fifteen minutes, and are repeated daily. As improvement goes on the length of time is shortened to three or four minutes. In all the cases the intolerable itching was quickly relieved, and the dermic infiltration reduced. In case of relapse the massage was again applied, and was employed as a preventive of future attacks. Two or three weeks, the author claims, were sufficient to cause complete disappearance of the prurigo. When complicated by eczema, the rubbing seemed to exert a beneficial effect on the latter also.—*Archiv. f. Derm. und Syph.*, 1893, XXV., p. 931.

---

### TREATMENT OF CERTAIN FORMS OF ACNE ROSACEA.

Petrini (*La Roumanie Med.*, 1893, No. 3), after ineffectual employment of all possible (?) procedures, finally adopted the following, with best results, in the case of two young persons. Pustules were emptied by Vidal's scarification method. Three days after each other all affected places were painted with

| | |
|---|---|
| Flexible collodion | 30 parts. |
| Icythyol | 2 part. |
| Resoricn | 1 part. |

Five or six days after the first painting the redness begins to disappear and the *acne* papules to decline. Recovery occurs usually after two or three repetitions of treatment.—*M. fur Prak. Derm.*, XVIII., *No.* 10.

### TREATMENT OF FURUNCLE.

Leloir (from *Rif. Med.*, 1893, No. 264) proposes the following:

1. Spraying with "carbolic acid solution" from a quarter to half an hour.

2. Drying with an absorbent, and then wetting with "salol-alcohol" or "iodoform-ether."

3. The application of a mercurial plaster composed as follows:

| | |
|---|---|
| Hg. et terpentin āā | 20 parts. |
| Emplast. diachyli | 50 parts. |
| Extract belladonna. | |
| Resina āā | 10 parts. |
| Acid salicyl | 6 parts. |

After twelve to twenty-four hours the plaster is removed and finally the furuncle is opened with the thermo-cautery, dressed with iodine or iodoform-ether, the opening being filled with salol or iodoform.—*M. für P. D.*, XVIII., *No.* 10.

---

### TREATMENT OF ACUTE ABSCESSES.

J. Aikmann (Glasgow *Med. Journ.*, 1893, No. 6) recommends the procedure of Barker: Deep incision, scraping out, then suturing. Rapid healing of a mammary abscess was thus obtained. The incision must be deep in order to release the accumulated serum in the surrounding tissues.—*M. für. Prak. Derm.* XVIII., *No.* 10.

A similar treatment is especially useful in infiltrated, suppurating, sebaceous glands in *acne indurata.—Ed.*

---

### ARSENIC IN RECURRENT HERPES.

Hutchinson is of the opinion that in this drug we have a means of controlling successive attacks of herpes. He gives a record of a case of "herpetiform pemphigus" in which formation of blebs was completely arrested by exhibition of arsenic. The disease returned when treatment was intermitted, but a continuance for two years produced a practical cure.—DR. JOHNSTON, in *Journal of Cutaneous and Genito-Urinary Diseases*, June, 1894.

